# Where Do Proteomics People Come from?

**DOI:** 10.1021/jasms.6c00268

**Published:** 2026-09-24

**Authors:** Benjamin C. Orsburn

**Affiliations:** † Organ Pathobiology and Therapeutics Institute, Pittsburgh, Pennsylvania 15203, United States; ‡ Department of Pharmacology and Chemical Biology, 6614 University of Pittsburgh, Pittsburgh, Pennsylvania 15203, United States

**Keywords:** proteomics scientists, technical training, workforce development, graduate education

## Abstract

The field of proteomics is one of the fastest-growing scientific disciplines in the world today. Beginning in 2020, ambitious market predictions anticipated proteomics to more than double in market size in 4–6 years and to even surpass the well-established genomic sciences industry in 8–12 years. While the market is still growing, it has not come close to matching the initial projections. To better understand the root cause of this, the author has collected field surveys and direct interviews of over 200 scientists actively involved in proteomics research today. While ultimately incomplete, it is worth noting that over 68% of respondents received their technical training in proteomics during either their doctoral or postdoctoral training. In stark contrast, only 4.3% of students obtained training during their undergraduate education. Given the 5.7-to-9.4-year average cycle for traditional doctoral and postdoctoral training in the life sciences, it appears the limiting factor in growing the proteomics field may be linked directly to current practices in training a qualified workforce.

## Graphical abstract

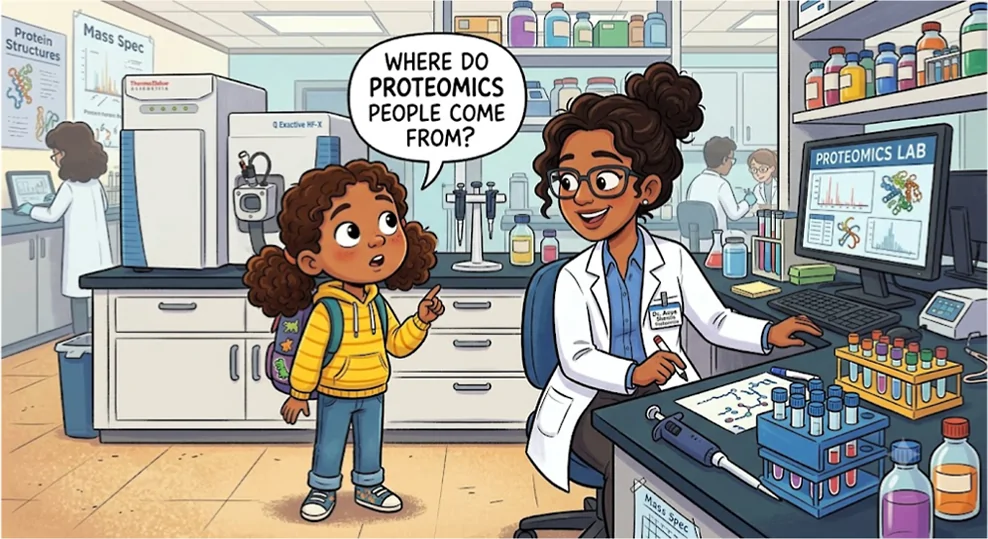

## Introduction

The term “proteomics” was first coined by Mark Wilkins in the late 1990s and was based on the recent increase in capabilities in the genomic sciences. Like “gene-omics”, “protein-omics” implies a measurement of all the proteins present in an organism, tissue, or cell.^1^ Unlike genomics, which has had the capacity to analyze nearly all of the approximately 30,000 human genes for decades, comprehensive measurement of the human proteome is fairly recent. It was not until 2017 when Bekker-Jennsen et al. first demonstrated a depth of coverage matching that of next-generation sequencing with 14,200 protein isoforms measured in a cell line in approximately one calendar day.^2^ Today, similar depth of coverage can be obtained in many sample types in up to 100 samples per instrument per day.^3^ In addition, new “next-generation” proteomics technologies have demonstrated the ability to selectively target thousands of proteins in human plasma, perhaps the most challenging of human materials.^4^

In late 2021, an article in Forbes magazine described the field of proteomics as “The Next Massive Investment Opportunity”.^5^ Optimistic market analyses the following year expanded on these hopes, with many groups estimating a rapid doubling of the value of the field. Some public analyses went as far as predicting proteomics to rapidly overtake the established, and still growing, genomics market. In 2026, it is obvious that the size of the field of proteomics has ultimately expanded, primarily in the pharmaceutical industry,^6^ but not to the level of those early market expectations.^7^

The rise of genomics in the early 2000s led to the rapid development of formal curricula in both undergraduate and graduate studies. By the early 2000s, it was possible to obtain a bachelor of science in genomics or genomic sciences from at least 40 universities in the United States (US). If you increase the breadth of this definition to undergraduate or graduate programs that offer an optional emphasis or focus on genomics, the number of institutions is easily over 120.^8^ Conversely, in four years of searching, the author has not identified a single undergraduate program focused exclusively on proteomics anywhere in the world.

This is strange if the two fields were equivalent in terms of the complexity of the challenge of analyzing their respective molecules. However, this is even more strange when the scope of the respective challenges is considered. Today it is well accepted that there are less than 30,000 human genes.^9^ In the simplest organisms or introductory textbooks, each of these genes leads to the production of a single protein. In reality, these numbers may be very different depending on how the protein is assessed. The HUPO Human Proteomics Project has been collecting evidence for predicted protein-coding regions of DNA for decades and currently catalogues 19,435 genes as having any evidence of protein-level expression, which is a far lower number than the Human Genome Project first predicted.^10^ However, many proteins have the function of modifying a certain group of proteins under specific conditions. These modifications can include cutting the produced protein to produce a shortened form, splicing two separate protein forms together, or adding chemical modifications such as a phosphate group or chain of sugars to the protein. Furthermore, other proteins may then modify just some of the proteins modified by the first set of modifying proteins under other, more specific, conditions. For example, a phosphorylated protein may be actively moved to another region of the cell where proteins are waiting to chemically modify those proteins that arrive.^11^ The effect of these modifications is typically not trivial. A phosphorylated protein, for example, may have a completely different function, activity, or location than the unmodified counterpart. Many versions of each protein may exist within each cell and may work in tandem or completely counteract the activity of other versions. This process of proteomic modification is not at all rare. There are easily more than 100,000 active protein versions, or proteoforms, in work in every one of us today, and the number may be far higher.^12^ The number of proteins is not the only thing that is higher. One metric of the relative value of measuring genes compared to proteins directly may be encapsulated in the respective number that has been effectively used as drug targets. Currently, over 90% of all drugs approved for use in the United States target specific proteins, rather than any intermediate molecule such as mRNA. More specifically, over 60% of all drugs target and/or inhibit specific isoforms of membrane-spanning proteins.^13^ From a cost-benefit perspective, it is no wonder that many drug development enterprises are skipping large-scale genomic studies and moving to proteomics first.^7^

If a scientific field is to double in total size, then it may not be necessary for the number of scientists within the field to double. The number of scientists would, however, absolutely have to increase. If there is no formalized education in the form of a degree for proteomics scientists, then the author thought to ask the obvious question. Where do experts in proteomics receive the training and skills that make them capable of accurately identifying and quantifying proteins in complex human samples? Or, more briefly, where do proteomics people come from?

## Results

### Survey Results

Of 226 proteomics researchers surveyed or researched from background interviews, 212 (93.8%) could be lumped relatively cleanly into a few categories based on the source of their training in proteomics. The final categories included undergraduate, master’s, doctorate, postdoctoral, or on-the-job training. A final category for “other” was necessary to encapsulate researchers who stated that they received their training from reading scientific literature or online content such as blogs or online videos. These have been summarized in Figure 1 and Supplemental Table 1. Over half (52.2%) of all scientists in this survey who are in the proteomics field learned proteomics during a Ph.D. or M.D./Ph.D. program. An additional 16.66% of scientists received their training during postdoctoral fellowships or research. On-the-job training is the third most common source of proteomics expertise today with 13.1% of responses. While some of these scientists likely learned from experts at their place of work, many also likely received vendor-specific training that was included or purchased along with analytical hardware. Undergraduate- or master’s-level training in proteomics, by contrast, is relatively rare, with 4.3% and 7.5% of responses, respectively. To put this in perspective, nearly 69% of all proteomics researchers have the most lengthy and advanced training in the academic sciences with at least a doctorate and many beyond that, with years of postdoctoral training. The level of this disparity in training is further amplified when the specific source is explored. While the results in Supplemental Table 1 have been anonymized for readers, they have not been for the author. The author feels it does no harm whatsoever to share that every proteomic scientist who was trained during their undergraduate degree received this instruction at Brigham Young University in Provo, Utah. Similarly, no researcher who received training during their master’s degree received this training in the US except for two students who worked with this author.

**Figure 1:**
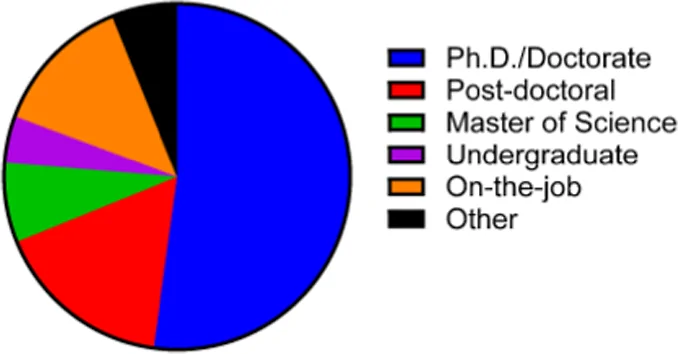
A summary of all survey respondents on where they received their initial training in proteomics.

### Training Length

According to the 2023 National Center for Education Statistics, the average length of a Ph.D. in the life sciences is 5.7 years from program start. This is generally after the completion of a bachelor’s degree, which still averages approximately 4 years. Postdoctoral fellowships in the US in the life sciences average an additional 3.7 years. This suggests that the minimum amount of time necessary to create a new proteomics scientist requires 9.7 years of undergraduate and graduate training or 13.4 years of education if postdoctoral training was also received.^14^

### Life Science Workforce Metrics

A comprehensive and recent analysis of the US life science workforce is encapsulated in the 2020 joint publication from the National Academies of Sciences, Engineering, and Medicine work “Safeguarding the Bioeconomy”.^15^ In this work, “Bioeconomy” refers to all branches of the agricultural, pharmaceutical, and biological sciences that contribute to the overall US economy.

While the difficulty in counting the number of workers in the life sciences is highlighted by these authors, it appears obvious that increasing the size of the bioeconomy requires increasing the number of available scientists in that field. The author would argue that the doubling of the size of the US bioeconomy from 2000 to 2014 was directly linked to the 160% increase in Ph.D. graduates in the life sciences over this same approximate period of time.^15^

## Discussion

Proteomics, in any form, is still a young field. Many of the scientists who invented the core techniques that make it possible are still researching and leading programs today. For example, Professor John Yates leads active research and one of the most respected journals in the field of proteomics. His Ph.D. thesis, “Protein sequencing by tandem mass spectrometry”, is a seminal work that framed the opportunities and challenges that we still face today. His groups later went on to develop critical technologies such as online two-dimensional fractionation^16^ and the first search engine for protein mass spectral data.^17^ While few lab leaders today have played such pivotal roles in the development of this, or any field, many had such significant parts that it is difficult to identify where they truly obtained their technical training. The author feels it is unlikely, however, that Dr. Yates will apply for a job at your company when you need to expand your proteomics discovery pipeline. With many experts happily entrenched in their careers, this leaves the question of how a field that analysts believe could double in size will obtain the scientific workforce that can perform these experiments. If trainees require a minimum of 5.7 to 9.4 years of training after completing their undergraduate degrees to contribute meaningfully to proteomics experiments, it seems like doubling the number of workers would require a minimum of that amount of time if we all started working on it today. In the hypothetical situation where the author and every other academic proteomic researcher did recruit twice as many students in the fall of 2026, we would undoubtedly increase the number of qualified proteomics scientists. But would that be enough?

For an extreme example, Emeritus Professor Don Hunt of the University of Virginia is responsible for training many of the most respected scientists in the field of proteomics today. When the author introduced Don at an invited lecture in Washington, DC, in 2017, the author said that he had mentored 123 Ph.D. students, and he did not correct the author. If that is the benchmark for success, and it should be, and there are international awards named for Don Hunt, is training more Ph.D. students even possible? At this point in the author’s career, if the author hoped to match Don’s number of Ph.D. students with a possibly manageable 10 students at a time and they all required the median 5.7 years to completion, the final batch would graduate right around the author’s 109th birthday, which would set back the author’s current retirement plans by more than 40 years. Possible? Maybe, but certainly not practical.

## Conclusions

The author started this multiyear off-and-on-again investigation to better understand the author’s own field. And the author thinks to have an answer to where current experts in the field get their training. Most proteomics people have a very similar story to the author’s, where they were in the right place at just the right time. They were lucky enough to study under some rare expert somewhere for a Ph.D. and eventually specialized in analyzing proteins. Again, like the author, many went on to do postdoctoral work in proteomics and found a fantastic job market that was hungry for their skills. However, if they are to grow this field to become the next genomics,^18^ they may need to think about revolutionary new avenues for training the next generation. The author is encouraged to see multiple recent reviews that are essentially textbooks on the field,^19^ and not only because it means the author will not have to go to the trouble of updating some books the author once wrote.^20^ In addition, they have seen some educational publications that have focused on implementing and outcomes^21^ of proteomic training.^22^ The author does not know what the answer is, but the author has sometimes thought that the author might be able to measure a protein concentration by fluorescence or clean a metal lens the author took out of a vacuum chamber without the author’s 12 years of combined undergraduate, graduate, and postdoctoral training.

### Study Limitations

The obvious limitation of this study is the sample bias. Not every proteomics scientist is on the social media platforms that the author knows how to use. It is highly possible that the ones that would see a survey the author posted are within some branch of the author’s social network that is biased toward scientists who are in academic-type research and enriched for researchers with longer educational routes.

## Materials and Methods

### Social Media Surveys

Between the summer of 2023 and the spring of 2025, the author twice posted on social media services, such as LinkedIn and BlueSky, a request for proteomics experts to self-report the source of their proteomic training. The text of these surveys was downloaded, and the results were painfully extracted manually into an Excel spreadsheet. As the results were fully anonymized, no IRB was obtained or required for this study.

### Expert Interviews

The US Human Proteomics organization posts interviews of proteomics experts on their website (www.us-hupo.org). The text from the US HUPO “Popstars”, as well as the handwritten notes from guests featured on The Proteomics Show podcast, was scraped by the author with his big heavy bifocals to extract training information for each interviewed expert. Following redundancy filtering by name in Excel, approximately 60 additional expert backgrounds were added to the final spreadsheet.

### Figure Generation

Figure 1 was generated by copy/pasting the results of the survey into GraphPad 11 using the “parts of a whole” option. The figure was then manually checked to find the most abrasive possible color scheme. The abstract graphic was generated by typing into Google’s search bar, “can you find me a way to make an awful AI image?”. When this led the author to a Gemini prompt, the author typed the request “Make a cartoon image of a child asking a scientist “where do proteomics people come from?”, and the image appeared in whatever version of Chrome the author did not authorize to be installed on the author’s office PC that was present on July 7th of 2026. After checking the image had illustrated cartoon humans with the correct number of eyes and fingers and that nothing overtly offensive was present in the image, the author copy-pasted it into this document. The image was later reloaded into multiple reverse image-based tools to determine whether it contained any protected content with no positive results. The author of this manuscript, therefore, takes full responsibility for the image as the sole mammalian contributor.

## Supplementary Material



## Notes

Supplemental Table 1—an Excel file with anonymized responses used in the creation of Figure 1 (XLSX)
